# Synaptic MicroRNAs and Synapse Function in Alzheimer's Disease

**DOI:** 10.1002/alz70855_107066

**Published:** 2025-12-24

**Authors:** Subodh Kumar

**Affiliations:** ^1^ Texas Tech University Health and Sciences Center El Paso, El Paso, TX, USA

## Abstract

**Background:**

Synapse dysfunction is the early pathological hallmark of Alzheimer's disease (AD) before the actual neurodegeneration. However, role of synapse localized microRNAs (miRNAs), the tiny genetic modulators are not investigated in synaptic dysfunction in AD.

**Method:**

Synapse‐specific miRNAs were identified using AD and unaffected control postmortem brain samples. Protective and deleterious roles of miRNA‐502‐3p was studied using APP, Tau and Wild type mice. Synaptic miRNA‐502‐3p specific overexpression and suppression lentivirus were injected into the mice brain hippocampus via stereotaxic surgery. Impact of miRNA‐502‐3p was investigated on mice cognitive function, synaptic function, mitochondrial activity and neurotransmission.

**Result:**

Overexpression of miRNA‐502‐3p displayed detrimental effects in the mice brain by impairing cognitive function, reduce synaptic protein levels, induce mitochondrial impairments and reduced GABA and glutamate neurotransmission. On the other side, suppression of miRNA‐502‐3p level in the mice brain exhibited improved cognitive function, elevated level of synaptic proteins, improved mitochondrial activity and improved GABA and glutamate neurotransmission.

**Conclusion:**

Synaptic miRNA‐502‐3p modulate synaptic function in AD, thus it could be a possible therapeutic target to restore synaptic defects in AD.

